# Are Teachers Working with Visually Impaired Children Prepared to Be Advocates of Oral Health? Pilot Study

**DOI:** 10.3390/children10071235

**Published:** 2023-07-18

**Authors:** Renata Chałas, Wioletta Bronislawa Mikuľáková, Paweł Maksymiuk, Agnieszka Skawińska-Bednarczyk, Lucia Hudáková, Justyna Pietrak, Ľudmila Andraščíková, Joanna Zubrzycka, Daniel Jordán, Andrea Radácsi, Judit Szőke

**Affiliations:** 1 Department of Oral Medicine, Medical University of Lublin, Chodźki 6, 20-093 Lublin, Poland; pawelmaksymiuk@umlub.pl; 2 Department of Physiotherapy, Faculty of Health Care, University of Prešov, Partizánska 3851/1, 080 01 Prešov, Slovakia; wioletta.mikulakova@unipo.sk; 3 Chair and Department of Peadiatric Dentistry, Medical University of Lublin, Chodźki 6, 20-093 Lublin, Poland; agnieszkaskawinskabednarczyk@umlub.pl (A.S.-B.); justynapietrak@umlub.pl (J.P.); 4 Department of Dental Hygiene, Faculty of Health Care, University of Prešov, Partizánska 3851/1, 080 01 Prešov, Slovakia; lucia.hudakova@unipo.sk (L.H.); ludmila.andrascikova@unipo.sk (Ľ.A.); daniel.jordan@unipo.sk (D.J.); 5 Preclinical Dentistry Lab, Medical University of Lublin, Chodźki 6, 20-093 Lublin, Poland; joannazubrzycka@umlub.pl; 6 Department of Dentistry, Oral and Maxillofacial Surgery, Medical School and Clinical Center, University of Pecs, Szigeti út 12, 7624 Pecs, Hungary; radacsi.andrea@pte.hu; 7 Department of Dentistry, Semmelweis Medical University, Üllői út 26, 1085 Budapest, Hungary; szoke.dr@t-online.hu

**Keywords:** oral health, visually impaired children, prevention, oral hygiene

## Abstract

Background: Children and adolescents with visual impairment are at increased risk of oral cavity diseases. Pro-health education in their prevention and the role of educators and school counselors are extremely important in this aspect. The aim of the study was to collect information, and compare and analyze the level of pro-health awareness in the field of oral health prevention among teachers working with visually impaired children in Poland and Slovakia. Methods: The questionnaire survey covered 109 school educators working with visually impaired children. The survey contained general information about participants concerning their knowledge of oral health, basic information about oral hygiene, and children’s care needs in this area. The obtained results were statistically analyzed. Results: The level of knowledge about oral health was assessed by the majority of respondents as rather good (60.56%), 28.44% as very good, and 11.01% as middling. Teaching children about oral hygiene at school was declared by a majority of them and over half of the correct answers were given by only 48.42% of the respondents. Conclusions: It is advisable to intensify the oral cavity diseases prevention training of teachers working with visually impaired children and youth and there is a great need to organize and carry out educational campaigns in schools for them.

## 1. Introduction

According to the Vision Loss Expert Group (VLEG) of the Global Burden of Disease Study (GBD) there are 43 million blind people in the world; 295 million people experience moderate to severe vision impairment and 258 million people experience mild vision impairment. It is estimated that the number of blind children in the world is 2 million. In addition, 30 million experience moderate to severe sight loss and 58 million experience mild sight loss [1]. The most common causes of visual impairment in children are refractive errors, followed by amblyopia, retinal disorders, congenital cataract, and corneal opacities. Blindness is mostly caused by cataracts, glaucoma, and refractive errors [2]. Children and adolescents with visual impairment are at increased risk of oral cavity diseases [3,4,5]. Unfortunately, they also have to overcome more barriers in accessing dental treatment compared to healthy people and, as a result, they visit the dentists less often than they should [6,7]. Their pro-health education and the role of tutors and school counsellors are extremely important. Pro-health behaviors are shaped from an early age. Oral health is also influenced by such factors as: level of education, socio-economic status, genetic predisposition, lifestyle, as well as social and physical environment and access to health services [8]. Health education can be considered in terms of many aspects, e.g., medical, social, pedagogical, and even cultural. Staying healthy is a key concept in health education.

Most often, the concept of pro-health education is understood as providing and consolidating appropriate knowledge that affects the pro-health behavior of children, adolescents, and adults, and contributes to increasing their health potential. This also applies to health education in the field of oral hygiene. A huge role in this education is played by educators in educational institutions who work daily with children and young people, where they spend several hours each day. They have the opportunity to not only provide young people with knowledge related to the general core curriculum of education but also to educate them on a social and health-promoting level. The level of awareness and involvement of teachers is extremely important because they have the ability to pass on the appropriate knowledge to their charges [8].

The level of education in the field of oral hygiene depends to a large extent on the education acquired during the educational period [9]. Early school children are especially susceptible to shaping appropriate behavior. At school, teachers are their health authority, so the knowledge provided in educational institutions shapes their critically important health awareness. Acquired habits allow people to use this knowledge in practice. In addition, thanks to the professional oral hygiene instructions which are carried out during dental visits, patients gain practical knowledge in the field of the prevention of oral diseases [10]. The provided information should be related to lifestyle and health behaviors, such as adopting proper hygiene and eating habits and applying for regular dental visits. It is important that the knowledge provided is understandable for them and triggers the need for preventive measures [10].

Data from the literature indicate that the population of people with disabilities are more likely to be diagnosed with oral diseases compared to healthy people. The disabled population has a higher incidence of caries disease, inadequate oral hygiene, and the presence of malocclusion [11,12]. Sensory disabilities, as well as the numerous systemic diseases that often accompany it, have a major impact on the development of caries and periodontal disease. The dental treatment of people with disabilities, unfortunately, often amounts to emergency assistance for pain [13]. 

So, the problem of oral hygiene of children and adolescents with disabilities is a challenge not only for themselves but also for their teachers, parents, and guardians. Children living in family homes brush their teeth with the help of their parents or relatives, whilst children living in dormitories are assisted by the teachers and caretakers employed there [14]. The curriculum should allow children to acquire the necessary brushing skills and improve the competencies related to proper oral hygiene among the guardians and teachers of visually impaired children. Research has shown the effectiveness of health education conducted by teachers [15]. School education and pro-health activities are also expected by parents [16]. Teachers must have knowledge in this area in order to be able to effectively fulfil this aim [15]. The lack of basic training may limit the willingness of teachers to participate in educational programs dedicated for pupils, and, consequently, this has an impact on children’s oral health in the future [17,18]. The aim of the pilot study was to collect information, and compare and analyze the pro-health awareness in oral health among teachers working with visually impaired children in Poland and Slovakia. The research was inspired by the international project “Oral health education program for visually impaired children” supported by Visegrad Fund.

## 2. Materials and Methods

### 2.1. Study Design and Data Collection

All aspects of the project were conducted in line with the ethical principles and were submitted to and approved by the Medical University of Lublin Bioethics Committee (KE-0254/48/02/2023). 

All participant details remained confidential and were only used for the purpose of the study. Informed consent was required by the participants to ensure their understanding of the above. All responses were anonymous.

### 2.2. Subjects/Samples

The survey was carried out among 109 teachers working directly with children with visual disabilities who agreed to take part in the study. The research covered 90 educators in Poland, working in the Zofia Sękowska Special School and Educational Center for disabled children in Lublin, and 19 employees of the School for Blind Children in Levoča, Slovakia. The questionnaire was prepared based on previous experiences in this topic [19] and modified to this group of respondents. All proposed questions were first discussed with all co-authors who are experts in oral health. The structured questionnaire was designed in Polish and then translated into Slovakian and Polish using the forward–backward translation method. Questionnaires were pre-tested in both countries prior to the main survey. 

The survey contained general information about participants (sex, age, work experience in years) and, concerning knowledge of oral health, basic information about oral hygiene and children’s care needs in this area. Its task was to determine the knowledge of the respondents about oral health and methods of proper dental hygiene. In addition, it contained questions about the possibility and control of oral hygiene in care centers and schools. The study also included questions on fluoride prophylaxis at the institution. This anonymous questionnaire included multiple questions. Types of questions applied in the questionnaire were open and closed types.

It is of utmost importance to acknowledge that the present investigation constitutes a pilot study serving as a precursor to a pivotal investigation. Consequently, the outcomes obtained herein shall provide a succinct portrayal of the target population and function as a guidepost for subsequent research endeavors pertaining to the primary concern of the study.

### 2.3. Statistical Analysis

The obtained results were statistically analyzed. The values of the analyzed quantitative variables were presented using the mean value, median, lower and upper quartiles, minimum and maximum values, standard deviation, and qualitative variables using the frequency and percentage. The dependence of qualitative variables was checked with the chi-square test. The normality of the distribution of variables in the study groups was checked using the Shapiro–Wilk test of normality. The Mann–Whitney U test was used to examine the differences in the number of correct answers between the two groups, and the Kruskal–Wallis test between the three groups. A significance level of *p* < 0.05 was adopted, indicating the existence of statistically significant differences or relationships. Descriptive statistics were conducted to identify the relative levels of oral health knowledge among teachers working with visually impaired children.

The database and statistical research were conducted using Statistica 9.1 computer software (StatSoft, Kraków, Poland).

### 2.4. Optimal Response Pattern

In order to assess the correctness of the studied group’s knowledge on oral health behaviors, our own criteria (Optimal Response Pattern) were selected, containing seven questions from among those asked in the questionnaire. These were selected questions relating to individual health-promoting oral hygiene habits. Teachers were asked to specify:

1. What is the correct time for toothbrushing? (expected correct answer: 2 min)
2. Which toothbrush bristle hardness is correct for children? (soft)
3. What is the correct time to replace a toothbrush with a new one? (every three months or when bristle wear is visible)
4. Which brushing technique is recommended for children? (circular motion)
5. Is cleaning the tongue recommended for children? (yes)
6. What is the recommended standard level of fluoride in toothpastes for children? (1000–1450 ppm)
7. At which age do permanent teeth start to erupt? (6–7 years)

The results obtained were analyzed to compare the answers of the surveyed individuals in relation to the pattern of behaviors important for maintaining proper oral hygiene.

## 3. Results

### 3.1. Characteristic of the Study Group

The examined teachers were aged between 23 and 64 years with a mean age of 44.25 ± 10.25 years. The group of respondents consisted of 89 women (81.65%) and 16 men (14.77%). A total of four teachers (3.67%) did not answer this question. The obtained education level was a master’s degree for 100 persons (91.74%), a bachelor’s for four teachers (3.67%) or high school level for five teachers (4.57%). The mean period of working as a teacher was 16.66 ± 10.07 years. A total of 33.02% of the group (36 teachers) had up to 10 years of work experience, 34.86% (38 people) between 11 and 20 years, and 29.36% (32 people) more than 21 years. An amount of 2.75% (three teachers) did not answer this question. The majority of teachers (82 persons, 75.23%) had their own children, 25 people (22.94%) did not, and 2 teachers (1.83%) did not reply to this question.

### 3.2. Self-Assessment of Knowledge on Oral Health

The self-assessment about knowledge in oral diseases prevention was, in the majority of the respondents, indicated as rather good (60.56%), 28.44% of the teachers considered that they had very good knowledge, and 11.01%—an average. In the group of teachers from Poland, 26.67% assessed their level of knowledge as very good, 62.22% as rather good, and 11.11% of respondents from this group indicated the answer “average”. The teachers from Slovakia answered, respectively: 36.84% assessed their level of knowledge about the prevention of oral diseases as very good, 52.63% as rather good, and 10.53% as average. The differences noticed between teachers indicate that higher numbers of educators having very good self-assessment work in Slovakia.

Teaching children about oral hygiene during classes at school was declared by 91.57% of respondents in PL and 100% in SK.

Of the respondents who assessed their knowledge at a very good level, 93.55% educated their pupils, and 84.62% of teachers assessing their level of knowledge as “good/average” passed on their knowledge. This relationship difference is not statistically significant (*p* > 0.05).

### 3.3. Professional Oral Health Education and Fluoride Prophylaxis Taking Place at School

Oral hygiene educational campaigns at school were reported by 92.66% of the respondents (98.84% among Polish teachers, 84.21% among Slovak teachers). The differences between countries were statistically significant (*p* < 0.05).

The respondents declared that oral hygiene educational campaigns were most often carried out by a nurse (49.54% of answers) or a school employee (38.53%), as well as fluoride prophylaxis educational action (nurse 37.61%, school staff 11.93%). Between both countries, there were statistically significant differences between people carrying out preventive actions (*p* < 0.001). In Slovakia, they were most often performed by a dental hygienist (73.68%), and in Poland by a nurse (57.78%).

Professional fluoride prophylaxis at school was reported by 98.55% of the Polish teachers and 92.86% of the Slovak teachers. The differences between countries were not statistically significant (*p* > 0.05).

Fluoride prophylaxis was most often carried out in schools in the form of drops/wipes (44.95%) and the use of gels (41.28%). No responders in either country indicated the use of fluoride tablets. In Poland, the gel was used statistically significantly more often than in Slovakia (PL 47.78%, SK 10.53%, *p* < 0.001). The use of drops/wipes was more often indicated by the Slovakian (63.16%) than the Polish (41.11%) teachers, without statistically significant difference (*p* > 0.05).

The frequency of educational actions concerning the prevention of oral diseases was defined by the majority of teachers as once a year (36.70%) and every 6 months (32.11%). There were statistically significant differences between the two countries (*p* < 0.05). Among teachers from Poland, the most frequently chosen answer regarding the frequency of educational actions was 6 months (44.87%). Among the answers of teachers from Slovakia, the vast majority selected once a year (over 83.33%).

The frequency of professional fluoride prophylaxis was defined by all teachers most frequently as every 6 months (32.11%). There were statistically significant differences between the two countries (*p* < 0.05). Among teachers from Poland, the most frequently chosen answer regarding the frequency of fluoride prophylaxis was 6 months (47.30%). Among the answers of teachers from Slovakia, the vast majority selected once a year (63.64%).

A total of 100% of the Slovak responders believed that oral hygiene educational campaigns among school teachers would contribute to improving the oral health of children, whereas only 87.84% of Polish teachers agreed with this sentence (*p* > 0.05).

### 3.4. Meals Consumed by Children and Toothbrushing at School

Both the Polish and Slovak respondents showed that most children (PL 98.89%, SK 100%, *p* > 0.05) ate meals at school. There was no statistically significant differences between countries for breakfast, second breakfast, and lunch (in Poland: breakfast 58.89% second breakfast 74.44%, and lunch 88.89%, while in Slovakia: breakfast 84.21%, second breakfast 68.42%, and lunch 94.74%, *p* > 0.05). 

The results differed significantly for the two countries in terms of toothbrushing. A total of 100% of Slovak responders confirmed that the children brush their teeth at school, while only 55.56% of Polish responders confirmed this (*p* < 0.001).

There were no statistically significant differences between countries for breakfast, second breakfast, and lunch.

In Poland, 9.00% of participants responded that children brush their teeth after breakfast, after second breakfast—16.67%, and after lunch—11.11%. In Slovakia, these results were 52.63% after breakfast, 0% after second breakfast, and 15.79% after lunch (*p* > 0.05).

In Slovakia, group tooth brushing was most often carried out after the midday meal (89.4%) and breakfast (over 52%), while, in Poland, it was most often carried out after lunch (16%). A total of 44% of children in Poland did not participate in brushing their teeth together at school after any meal, while all children in Slovakia participated in group tooth brushing (*p* < 0.001).

Totals of 89.89% of respondents believe that oral hygiene educational campaigns among school teachers would contribute to improving the oral health of children (PL 87.84%, SK 100.00%, *p* > 0.05).

### 3.5. Optimal Response Pattern

Basic information about methods of proper oral hygiene, in order to determine the knowledge of the respondents about oral health, were checked in seven of the questions described in the Materials and Methods section.

Table 1 shows the descriptive statistics of the number of teachers’ correct answers.

More than half (51.38%) of the surveyed teachers correctly answered only three questions on the prevention of oral diseases (Table 2).

A statistical analysis was performed to assess the differences in the numbers of correct answers between teachers based on specific variables (Table 3).

There was no statistically significant difference in the number of correct answers in the survey between the respondents who assessed their knowledge as very good or rather good/middling (*p* > 0.05). The mean percentage of correct answers in the group who assessed their knowledge as very good was 49.31%, whereas the group who assessed their knowledge at a lower level (rather good, middling) scored a mean of 50.92%.

There was no statistically significant difference in the number of correct answers in the survey between the respondents who declared that they pass on their knowledge about oral hygiene to the pupils and those who did not provide such knowledge (*p* > 0.05). The mean percentage of correct answers in the group who passed on their knowledge was 52.03%, whereas the group who did not pass on their knowledge scored a mean of 39.80%.

There was no statistically significant difference in the number of correct answers in the survey between the respondents who declared that they have their own children and those who declared that they do not (*p* > 0.05). The mean percentage of correct answers in the group with children was 49.47%, whilst in the group without it was 53.71%.

There was no statistically significant difference in the number of correct answers between males and females (*p* > 0.05). The mean percentage of correct answers in the male group was 51.79%, whilst in the female group it was 49.76%.

The correlation of correct answers to the questions regarding work experience was also analyzed. There was no statistically significant difference in the number of correct answers between the respondents from the three compared seniority groups (*p* > 0.05).

## 4. Discussion

Health is one of the most important values in human life, depending on many factors, and to a large extent on the preferred pro-health behaviors. Pro-health education plays a leading role in the process of pro-health behaviors education [15]. Pro-health education on the prevention of oral diseases is an effective tool in increasing awareness, knowledge, attitudes, and actions in preventing oral cavity diseases [16].

The correlation between oral health and health-promoting habits regarding hygiene and diet is well documented in the literature [5,6,7,8]. Nowadays, in dentistry, the need for preventive (prophylactic) actions is clearly recognized [20]. Numerous studies indicate the poor oral health of children and adolescents in many countries around the world [3,6,9,15,18]. This may be the result of insufficient oral health educational programs addressed to teachers, parents, and guardians [14].

People with visual impairment often have to deal with numerous difficulties in everyday life. Maintaining proper oral hygiene is one of many tasks that is much more difficult than for those with normal vision [13].

The unsatisfactory oral health level of the visually impaired children is shown in numerous research studies [6,9,20]. This could be the result of the lack of preventive state programs for children with visual impairment and parents’ low dental awareness level. The problem could also lie in insufficient care regarding oral hygiene previously in the kindergartens. They report not brushing teeth after each main meal, the sparsely or ineffectively executed fluoride programmes and educational actions, a lack of hygienic aids such as dental floss or mouth rinses among children, a lack of parent/caregiver assistance during daily toothbrushing, and the same is continued in schools now [20,21,22,23]. 

Familiarizing the pupils with knowledge about health, shaping pro-health habits, and creating effects and activities for health are some of the basic tasks that a teacher should perform. The school’s effectiveness in this depends on many factors: the teacher’s work, their actions, knowledge, and skills [15]. It has been proven that the effects of teacher training in proper oral hygiene and the subsequent education of charges in this area always give very good results. The oral hygiene of the patients is improved and the indicators of caries activity are reduced [10,18].

In our preliminary study, almost all examined teachers confirmed passing on their knowledge to visually impaired children regarding oral cavity health, but we found their knowledge on proper oral hygiene to be unsatisfactory. This indicates the need for educating teachers so that, when they themselves are aware of the role of oral health and of the methods for its maintenance, they can pass on this knowledge to students. But, due to the small number of respondents, this survey should be treated as a pilot study. Nevertheless, our findings highlight the importance of providing adequate educational resources in order to raise awareness and knowledge on the topic, and to promote the continued education on the matter for both teachers/parents and their visually impaired children. It is advisable to disseminate pro-health education among children and young people, as well as among educators in special schools and kindergartens for visually impaired children and in integration classes. Actions should also be taken to increase the sensitivity of society to the needs of visually impaired children [2,10].

The obtained results indicate the need to continue the research. They also show that, although the general knowledge of teachers about the principles of caring for children’s oral health is positively self-assessed, it is still insufficient to conduct school education in this area [22,23]. We see room for special education tools which would be very helpful both for teachers and children, like tooth models with Braille descriptions, leaflets, and sound records. This could encourage educators to actively use them in oral health education.

On the other hand, we can self-critically state that the knowledge of dental professionals is also lacking in this field. So, it would also be important for dentists, chairside assistants, and dental hygienists to learn the special pedagogical methods that can be effectively applied to visually impaired children during oral hygiene education sessions.

## 5. Conclusions

The conducted research indicates the fact that the majority of teachers at schools for visually impaired children have general, but not expert, knowledge of oral health. Most of them believe that broader knowledge would correlate with improvement in their blind pupils’ oral health status. 

It is advisable to intensify the oral cavity disease prevention training of teachers working with visually impaired children and youth, and to organize and carry out educational campaigns in schools. It appears that trained teachers could play an important role in raising children’s health status, which would provide measurable benefits, both financially and medically. 

According to experts on this field, tendency on visual impairment may aggravate, so dental prevention/pro-health education should be more emphasized because more and more oral health educators are and will be needed.

## Figures and Tables

**Table 1:** Descriptive statistics of the number of teachers’ correct answers.

	N	Mean	Median	Min	Max	Q1	Q3	SD
Correct answers (number)	109	3.53	3.0	0.00	7.00	3.00	5.00	1.38
Correct answers (percentage)	109	50.46	42.86	0.00	100.00	42.86	71.43	19.69

**Table 2:** Teachers’ correct answers in a questionnaire.

	Correct Answers	
	Number of Teachers	%
0–3 correct answers	56	51.38
4–5 correct answers	46	42.20
6–7 correct answers	7	6.42

**Table 3:** Descriptive statistics and *p*-value of the percentage of correct answers in the analyzed groups.

Analyzed Variables		M	Me	Min	Max	Q1	Q3	SD	Comparison of Averages
Subjective assessment of knowledge	very good/	49.31	42.86	14.29	85.71	28.57	71.43	19.81	Z = −0.537 *p* = 0.592
	rather good/middling	50.92	50.00	0.00	100.00	42.86	71.43	19.75	
Transfer of knowledge to children	yes	52.03	57.14	14.29	100.00	42.86	71.43	18.86	U = 472.5 *p* = 0.081
	no	39.80	42.86	0.00	71.43	28.57	57.14	22.54	
Having their own children	yes	49.48	42.86	0.00	100.00	28.57	71.43	1.43	Z = −0.867 *p* = 0.386
	no	53.71	57.14	28.57	85.71	42.86	71.43	17.63	
Sex	female	49.76	42.87	0.00	100.00	28.57	71.43	20.56	U = 667.5 *p* = 0.694
	male	51.79	50.00	28.57	71.43	42.86	71.43	16.39	
Work experience	<10 years	50.00	42.86	14.29	85.71	35.71	64.29	18.23	H = 4.324 *p* = 0.115
	11–20 years	55.26	57.14	28.57	85.71	42.86	71.43	17.63	
	>21 years	45.09	42.86	0.00	100.00	28.57	57.14	22.69	

## Data Availability

Not applicable.
